# Acquired laryngomalacia as a cause of post-extubation stridor and extubation failure following craniotomy: a case report

**DOI:** 10.1186/s40981-023-00637-5

**Published:** 2023-07-15

**Authors:** Kazuyuki Mizunoya, Keisyu Onodera, Yuki Takahashi, Takayuki Toki, Hitoshi Saito, Yuji Morimoto

**Affiliations:** 1 Department of Anesthesiology and Critical Care Medicine, Hokkaido University Hospital, N14W5, Kita-Ku, Sapporo, 060-8648 Japan; 2 Department of Emergency Medicine, Hokkaido University Hospital, N14W5, Kita-Ku, Sapporo, 060-8648 Japan

**Keywords:** Acquired laryngomalacia, Post-extubation stridor, Epiglottis, Arytenoid

## Abstract

**Background:**

Laryngomalacia is a dynamic airway condition characterized by flaccid laryngeal tissue and inward collapse of supraglottic structures during inspiration. Although it may cause airway obstruction and requires careful management, there have been few reports regarding laryngomalacia after surgery. We report a case of adult-onset laryngomalacia occurred after craniotomy requiring reintubation.

**Case presentation:**

A 21-year-old man was admitted to the ICU after craniotomy for a cerebellopontine angle tumor. He developed severe stridor immediately after extubation on the postoperative day 2 and required reintubation. On the postoperative day 5, similar episode occurred following re-extubation and fiberoptic laryngoscopy revealed a collapsed epiglottis and left arytenoid into the glottis. A diagnosis of laryngomalacia was made, and he underwent tracheostomy. Laryngomalacia had completely improved; however, bilateral vocal cord paralysis was detected 2 weeks later.

**Conclusions:**

Acquired laryngomalacia should be considered as a possible mechanism of the airway symptoms in a patient with neurological dysfunction.

**Supplementary Information:**

The online version contains supplementary material available at 10.1186/s40981-023-00637-5.

## Background

Laryngomalacia is a dynamic airway condition characterized by inward collapse of flaccid supraglottic structures during inspiration [1]. Most cases of laryngomalacia occur in infants with congenital abnormalities and are the most common cause of stridor in children [2]. In contrast, adult-onset laryngomalacia is a rare condition and only a few case reports have been published [3]. Decreased neuromotor tone of the pharynx due to neurological injuries or surgical resection of the suprahyoid muscle group are common etiologies in patients with acquired laryngomalacia [1]. However, there is only one case report of acquired laryngomalacia occurring in the acute postoperative phase of elective craniotomy [4]. Here, we present another case of acquired laryngomalacia involving multiple regions, including the epiglottis and arytenoid, which caused repeated extubation failure after elective craniotomy.

### Case presentation

A 21-year-old man with no previous history underwent elective craniotomy for cerebellopontine angle tumor under general anesthesia with propofol and remifentanil. Because of intraoperative acute brain swelling due to intra-tumoral hemorrhage, the operation was completed after additional internal decompression of the left cerebellum and external decompression. After surgery, the patient was admitted to our intensive care unit to control intracranial pressure under deep sedation. Computed tomography performed on postoperative day (POD) 2 revealed decreased brain edema. After stopping propofol infusion, he fully awoke with a Glasgow Coma Scale score of E4VTM6 and was extubated without a cuff leak test. However, the patient rapidly developed inspiratory stridor and dyspnea with tracheal tagging and chest retraction following extubation. Insertion of a nasopharyngeal airway and suctioning of secretions did not improve his respiratory failure, and reintubation was performed because oxygen saturation deteriorated. Laryngeal edema evaluated using a videolaryngoscope was slight, so an endotracheal tube of the same size as the removed tube (inner diameter 8.0 mm) could be inserted smoothly. At that time, the cause of the stridor was unknown. Because surgical manipulation might transiently damage the lower cranial nerves and reduce laryngeal function, we decided to attempt re-extubation after several days of mechanical ventilation.

**Fig. 1:**
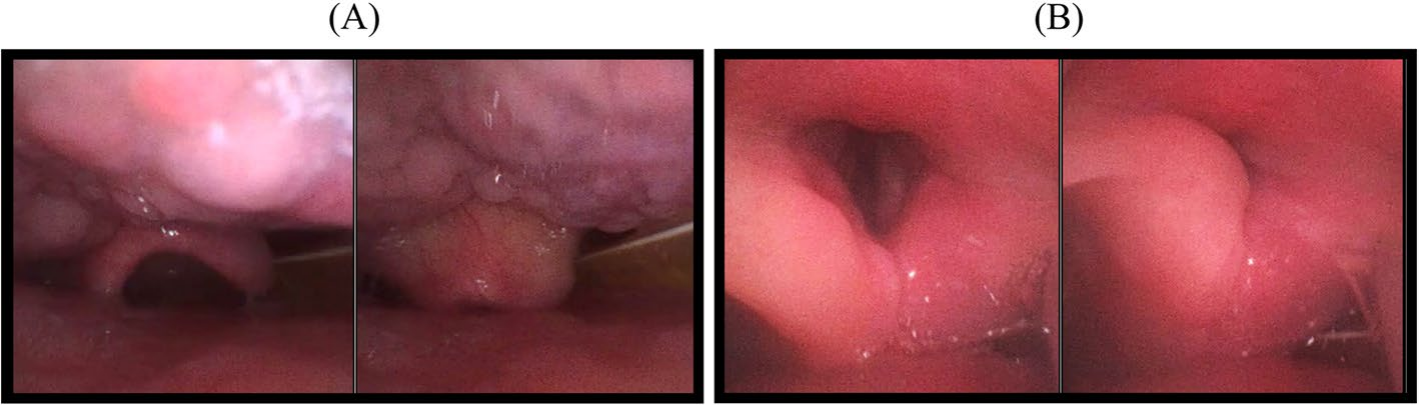
Fiberoptic laryngoscopy view of the pharynx at the time of the second extubation. In both **A** and **B**, the left panel represents expiration and the right panel represents inspiration. **A** The mildly edematous epiglottis prolapses downward during inspiration. **B** The left arytenoid mucosa is swollen and falls into the glottic inlet during inspiration

**Fig. 2:**
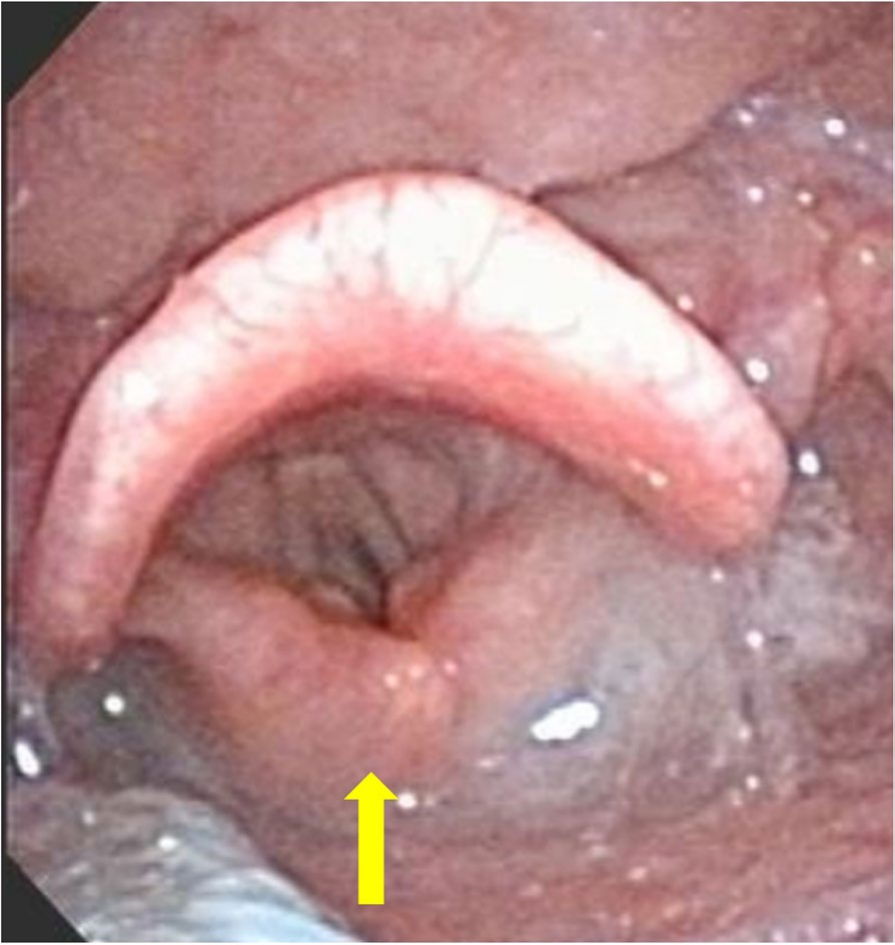
Fiberoptic laryngoscopy view of the larynx 2 months after craniotomy. Left arytenoid mucosa is redundant compared to the right (yellow arrow)

On POD-5, after confirming negative cuff leak test (leak volume < 110 mL), the patient passed spontaneous awakening and breathing trials and was re-extubated. As with the initial extubation, stridor recurred, and fiberoptic laryngoscopy was performed in Fowler’s position. It revealed mildly swollen supraglottic tissues and a downward collapse of the epiglottis during inspiration (Fig. 1A). Additionally, the left arytenoid was swollen unilaterally and was sucked into the glottic inlet during inspiration, resulting in severe airway obstruction (Fig. 1B and Supplemental Video 1). Based on these findings, acquired laryngomalacia was diagnosed as the cause of the patient’s stridor. Simultaneously, aspiration of oral secretions was observed, which led to even greater dyspnea, and the patient was intubated again. Since the short-term prospects for improvement of laryngomalacia and impaired laryngeal function are unclear, a tracheostomy was performed on POD-7. Laryngeal re-evaluation was performed by an otolaryngologist on POD-18. Although the laryngomalacia improved completely, redundant mucosa of the left arytenoid was observed, even after the swelling subsided. In addition, bilateral vocal cord paralysis caused by vagal nerve palsy due to aggressive resection of the tumor was observed, and airway management with tracheostomy was continued. Two months later, although the right vocal cord motion was gradually restored, the left vocal cord motion had not recovered and excess mucosa of the left arytenoid was still observed (Fig. 2).

## Discussion

Most cases of laryngomalacia are diagnosed during infancy. Although the exact mechanism underlying its development is unknown, congenital factors such as anatomical, cartilaginous, and neurological theories have been proposed [5]. In contrast, adult-onset laryngomalacia is thought to be caused by an acquired pathophysiological conditions such as central nervous system (CNS) insults, transcervical surgeries, and head or neck trauma [1]. Ferri et al. reviewed 41 cases of adult-onset laryngomalacia reported between 1960 and 2019 and found that neurological injury was the most commonly identified etiology [3]. The majority of reported neurological injury-related laryngomalacia cases involved either the epiglottis or the arytenoids/aryepiglottic folds, and only a few mixed-type cases have been reported [1].

Epiglottic prolapse is caused by impaired neuromotor tone in the supporting tissue of the epiglottis. Furthermore, pharyngeal hypotonia is believed to cause narrowing of the pharyngeal cavity and exacerbate epiglottic prolapse through the Bernoulli effect during inspiration [6]. In contrast, arytenoid/aryepiglottic fold-type laryngomalacia is thought to be related to redundancy in the arytenoid regions [7]. Although few cases of acquired laryngomalacia with neurological injury have been reported, the causal relationship between neurological disorders and arytenoid/aryepiglottic fold-type laryngomalacia remains unclear. In the present case, swollen arytenoid mucosa was sucked into the glottic inlet. Since fiberoptic laryngoscopy performed 2 months later also showed excess mucosa in the left arytenoid region, we hypothesized that the left arytenoid collapse was caused by edema of the originally redundant mucosa, in addition to impaired lower cranial nerves.

In this case, we could not consider laryngomalacia as the cause of post-extubation stridor (PES) at the time of the initial extubation. Although laryngeal edema is the most common cause of PES, the possibility of adult-onset laryngomalacia causing PES is poorly understood. However, acquired laryngomalacia should be differentiated in high-risk patients such as those with CNS insults or damaged lower cranial nerves. Fiberoptic laryngoscopy is a promising diagnostic tool for PES because it can visualize structural (e.g., edema) or functional (e.g., laryngomalacia and vocal cord paralysis) abnormalities in the laryngeal tissue. In particular, functional abnormalities are difficult to diagnose before extubation or after the administration of anesthetics and muscle relaxants. In previous reports, upper airway management such as nasopharyngeal airway insertion [8], jaw-thrust maneuver [4], and head elevation [9] have been reported to improve airway symptoms in epiglottis-type laryngomalacia. As acquired laryngomalacia may be overlooked in such cases, the cause of PES should not be neglected.

Neurological injury-related laryngomalacia has been reported to improve with recovery of neurologic function [10]. However, differences in the clinical course among types of laryngomalacia (epiglottic, arytenoid, or mixted) are unclear. In our case, the laryngomalacia resolved entirely on POD-18 and if it had not been for concomitant bilateral vocal cord paralysis, tracheostomy could have been avoided. Therefore, airway management should be considered according to the type of laryngomalacia, prospect of neurological recovery, and other concomitant airway complications.

## Supplementary Information

**Additional file 1: Video 1.** Fiberoptic laryngoscopy view of laryngomalacia in the arytenoid region.

## Data Availability

Not applicable.

## Abbreviations

POD: Postoperative day
CNS: Central nervous system
PES: Post-extubation stridor

## References

[1] Hey SY, Oozeer NB, Robertson S, MacKenzie K (2014). Adult-onset laryngomalacia: case reports and review of management. Eur Arch Otorhinolaryngol.

[2] Dobbie AM, White DR (2013). Laryngomalacia. Pediatr Clin North Am.

[3] Ferri GM, Prakash Y, Levi JR, Tracy LF (2020). Differential diagnosis and management of adult-onset laryngomalacia. Am J Otolaryngol.

[4] Akimoto Y, Bando N, Sato H, Sato K, Momota K, Nunomura T (2022). Acquired laryngomalacia as a cause of post-extubation respiratory failure in patient with postoperative seizure and central pontine myelinolysis after craniotomy. J Med Invest.

[5] Thompson DM (2007). Abnormal sensorimotor integrative function of the larynx in congenital laryngomalacia: a new theory of etiology. Laryngoscope.

[6] Woo P (1992). Acquired laryngomalacia: epiglottis prolapse as a cause of airway obstruction. Ann Otol Rhinol Laryngol.

[7] Peron DL, Graffino DB, Zenker DO (1988). The redundant aryepiglottic fold: report of a new cause of stridor. Laryngoscope.

[8] Takeshita J, Nishiyama K, Fujii M, Tanaka H, Beppu S, Sasahashi N (2017). Repetitive postoperative extubation failure and cardiac arrest due to laryngomalacia after general anesthesia in an elderly patient: a case report. J Anesth.

[9] Mima H, Ishida H, Yamazaki K (1996). Acquired laryngomalacia as a cause of airway obstruction immediately after unilateral mouth floor surgery. Anaesthesia.

[10] Wiggs WJ, DiNardo LJ (1995). Acquired laryngomalacia: resolution after neurologic recovery. Otolaryngol Head Neck Surg.

